# Estimating the Clinical and Economic Benefit Associated with Incremental Improvements in Sustained Virologic Response in Chronic Hepatitis C

**DOI:** 10.1371/journal.pone.0117334

**Published:** 2015-01-30

**Authors:** Phil McEwan, Thomas Ward, Hayley Bennett, Anupama Kalsekar, Samantha Webster, Michael Brenner, Yong Yuan

**Affiliations:** 1 HEOR Ltd, Singleton Court Business Park, Monmouth, Wales, United Kingdom; 2 Swansea Centre for Health Economics, School of Human & Health Sciences, Swansea University, Wales, United Kingdom; 3 Global Health Economics and Outcomes Research, Bristol-Myers Squibb, Princeton, New Jersey, United States of America; 4 Bristol-Myers Squibb, Uxbridge, Middlesex, United Kingdom; Centers for Disease Control and Prevention, UNITED STATES

## Abstract

**Introduction:**

Hepatitis C virus (HCV) infection is one of the principle causes of chronic liver disease. Successful treatment significantly decreases the risk of hepatic morbidity and mortality. Current standard of care achieves sustained virologic response (SVR) rates of 40–80%; however, the HCV therapy landscape is rapidly evolving. The objective of this study was to quantify the clinical and economic benefit associated with increasing levels of SVR.

**Methods:**

A published Markov model (MONARCH) that simulates the natural history of hepatitis C over a lifetime horizon was used. Discounted and non-discounted life-years (LYs), quality-adjusted life-years (QALYs) and cost of complication management were estimated for various plausible SVR rates. To demonstrate the robustness of projections obtained, the model was validated to ten UK-specific HCV studies.

**Results:**

QALY estimates ranged from 18.0 years for those treated successfully in fibrosis stage F0 to 7.5 years (discounted) for patients in fibrosis stage F4 who remain untreated. Predicted QALY gains per 10% improvement in SVR ranged from 0.23 (F0) to 0.64 (F4) and 0.58 (F0) to 1.35 (F4) in 40 year old patients (discounted and non-discounted results respectively). In those aged 40, projected discounted HCV-related costs are minimised with successful treatment in F0/F1 (at approximately £300), increasing to £49,300 in F4 patients who remain untreated. Validation of the model to published UK cost-effectiveness studies produce R2 goodness of fit statistics of 0.988, 0.978 and of 0.973 for total costs, QALYs and incremental cost effectiveness ratios, respectively.

**Conclusion:**

Projecting the long-term clinical and economic consequences associated with chronic hepatitis C is a necessary requirement for the evaluation of new treatments. The principle analysis demonstrates the significant impact on expected costs, LYs and QALYs associated with increasing SVR. A validation analysis demonstrated the robustness of the results reported.

## Introduction

Chronic hepatitis C represents a major public health burden due to the development of cirrhosis, which is associated with an increased risk of end-stage liver disease (ESLD) related morbidity and mortality [1]. In the UK, it is estimated that 214,000 people are currently chronically infected with the hepatitis C virus (HCV) [2]; of these, between 10% and 20% are likely to develop cirrhosis over the 20–30 year period following infection [3].UK hospital admissions for HCV-related ESLD complications have increased from 608 in 1998 to 2,390 in 2012, and HCV-related deaths have increased from 98 in 1996 to 428 in 2012 [2]. The slow progressive nature of HCV infection means that whilst the incidence of new infections has decreased over time [4], the incidence of late stage complications will continue to rise unless those infected are successfully treated. The accepted clinical endpoint of HCV treatment is the achievement of a sustained virologic response (SVR), defined as an undetectable level of HCV RNA at a certain time point after end of treatment (e.g. 12 [SVR12] or 24 weeks [SVR24]). EASL guidelines state that the primary goal of HCV therapy is to cure the infection, which is generally associated with resolution of liver disease in patients without cirrhosis [5]. Long-term follow-up studies have shown that an SVR corresponds to a definitive cure of HCV infection in more than 99% of cases [6]. Patients who achieve SVR are at significantly decreased risk of hepatic morbidity and mortality [7]. HCV genotype, genetic polymorphisms (IL28B) and the stage of liver fibrosis upon initiating treatment are the strongest predictors of the likely achievement of SVR [5].

Current standard of care (SoC) in the UK for HCV genotype 1 infection is triple therapy comprising peg-interferon alfa, ribavirin and a protease inhibitor (either boceprevir or telaprevir) [8,9]. For non-genotype 1 HCV infection, SoC is combination therapy with peg-interferon alfa plus ribavirin [10].

Rates of SVR vary between 40% and 80% depending on the treatment used, HCV genotype, disease stage and other patient characteristics [11]. The therapy landscape for treating HCV is rapidly expanding [11]. 2014 UK guidelines identify roles for the new direct acting antivirals (DAAs) daclatasvir, sofosbuvir and simeprevir in the management of chronic hepatitis C [12]. While these therapies are associated with higher SVRs, reduced treatment durations and fewer adverse events compared to SoC, concerns have been raised that the significant differences in the cost-per-cure for newer regimens may result in providers potentially favouring cheaper, less well tolerated regimens [12]. The emergence of new treatments adds additional choice and regimen complexity for clinicians and payers. Estimating the health economic consequences of competing therapies is generally undertaken using cost-effectiveness analysis. In the current HCV landscape, the possible permutation of such analysis is considerable. Cost-effectiveness is fundamentally influenced by treatment response, which is dependent upon factors such as genotype, fibrosis stage and prior treatment status, but also age, duration of therapy (due to either discontinuation or response-guided therapy) and treatment disutility. Consequently, aligning therapy- and patient-specific characteristics to ensure optimal value for money will become increasingly challenging. In order to offer insight into the potential clinical and economic benefit associated with treatment success in HCV, the principle objective of this study was to quantify the benefit that increasing levels of SVR would be expected to realise in terms of predicted life-years (LYs) gained, quality-adjusted life-years (QALYs) gained and costs of managing ESLD-related complications. As the credibility of these projections are dependent upon the robustness of the underlying model used, a secondary objective was to assess the validity and generalisability of the results, and therefore the model used, from a UK perspective.

## Methods

### Model

**Fig 1:**
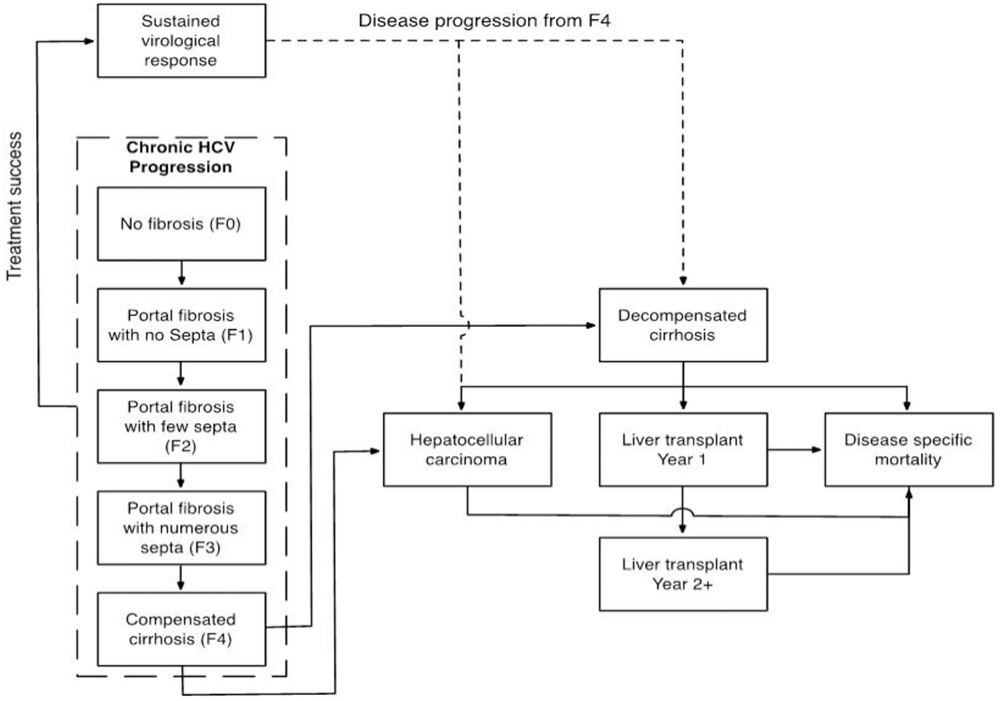
Flow diagram of the MONARCH model.

The ‘MOdelling the NAtural histoRy and Cost-effectiveness of Hepatitis’ (MONARCH) model was used to undertake this study. The MONARCH model has previously been described in detail [13–15]; in brief, the MONARCH model is a cohort-based Markov model designed to simulate the natural history of hepatitis C and its complications. The MONARCH model runs in annual cycles over a variable time horizon, up to patient lifetime. At entry, patient cohorts are distributed across fibrosis stages, defined using the METAVIR scoring system (F0–F4), from which they progress to ESLD complications (decompensated cirrhosis [DC], hepatocellular carcinoma [HCC] and liver transplant [LTx]) and death, or to a state of SVR if treatment is successful. Progression through fibrosis stages is modelled via the use of dynamic age- and fibrosis stage-specific transition rates drawn from a previously published meta-regression analysis [16]. Disease progression through ESLD complications is modelled using previously published transition rates, Table 1. The MONARCH model flow diagram is presented in Fig. 1.

**Table 1:** Default disease state transition rates applied in the model.

Transition	Mean	Distribution	Parameters		Source
			Alpha	Beta	
F0 to F1	0.079*	Normal			[16]
F1 to F2	0.073*	Normal			[16]
F2 to F3	0.111*	Normal			[16]
F3 to F4	0.051*	Normal			[16]
F3 to DC	0.000	Beta			
F3 to HCC	0.000	Beta			
F4 to DC	0.039	Beta	96.06	2,367.04	[44]
F4 to HCC	0.014	Beta	98.59	6,943.27	[44]
F4 post-SVR to DC	0.001	Beta	4.036	4032.054	[17]
F4 post-SVR to HCC	0.008	Beta	6.767	839.093	[17]
DC to HCC	0.014	Beta	98.59	6,943.27	[44]
DC to LTx	0.030	Beta	96.97	3,135.36	[44]
DC to death	0.130	Beta	86.87	581.36	[44]
HCC to LTx	0.040	Beta	95.96	2,303.04	[44]
HCC to death	0.430	Beta	56.57	74.99	[44]
LTx (Year 1) to death	0.210	Beta	78.79	296.40	[44]
LTx (Year 2+) to death	0.057	Beta	94.24	1,559.14	[44]

*Transition rates are influenced by the coefficients presented in Table 2; rates presented here are the mean values as reported in the original study.

Modelling antiviral therapy was undertaken using a decision tree that allows the characterisation of adverse event rates, discontinuation, timings of predictive tests, treatment success (i.e. achieving SVR), therapy and monitoring costs, and treatment disutility. Furthermore, the model was constructed to allow treatment profiles (e.g. effectiveness) to vary across fibrosis stages. Patients who achieve SVR from the compensated cirrhosis state (F4) were at an increased risk of developing HCC and DC [17], in line with previous studies [18–21]. Patients who achieved SVR from fibrosis stage F0–F3 were at the same risk of developing HCC and DC as the general population. In those subjects failing to respond to treatment, progression continues from the fibrosis stage they were in when commencing antiviral therapy. All-cause mortality rates were applied annually to patients who achieve SVR, remain chronically infected or develop compensated cirrhosis; for those progressing to more advanced ESLD, disease-specific mortality rates are applied. The default MONARCH transition rates are presented in Table 1 and regression coefficients for fibrosis stage progression equations are presented in Table 2.

**Table 2:** Log-linear regression equation coefficients used to derive age dependent fibrosis stage specific transition probabilities.

Transition	Coefficient	Mean	Standard error	Distribution
F0 to F1	Intercept	2.10400	0.664	Normal
	Duration	0.07589	0.011	Normal
	Design	0.32470	0.175	Normal
	Male	0.50630	0.478	Normal
	Genotype	0.48390	0.278	Normal
F1 to F2	Intercept	1.53870	0.818	Normal
	Duration	0.06146	0.014	Normal
	Excess alcohol	0.80010	0.391	Normal
F2 to F3	Intercept	1.60380	0.590	Normal
	Age	0.01720	0.012	Normal
	Duration	0.05939	0.010	Normal
	Excess alcohol	0.45390	0.280	Normal
F3 to F4	Intercept	2.28980	0.773	Normal
	Age	0.01689	0.015	Normal
	Duration	0.03694	0.013	Normal
	IDU	0.59630	0.316	Normal
	BT	1.16820	0.368	Normal
	Genotype	0.46520	0.291	Normal

Risk Factor	Description
Duration	Length of time from the presumed date of infection to the date of liver biopsy.
Design	Set to 0 if the study design is cross sectional and set to 1 if the study design is retrospective-prospective.
Male	Proportion of patients that are male.
Genotype	The meta-regression investigated the effect of having genotype 1 and genotype 2 or 3 infection. Set to 1 for genotypes 1a and 1b or set to 0 for genotypes 2 and 3.
Excess Alcohol	Defined as alcohol consumption of at least more than 20 g/day.
Age	Age at cohort initiation.
IDU	The proportion of individuals that acknowledged injection drug use (IDU) as the main risk factor for HCV progression.
BT	The proportion of individuals that were newly diagnosed with chronic hepatitis C at blood donor screening.

Source: All risk factors are described as in the source publication, Thein *et al*. [16].

Health states within the model were subject to specific costs, applied annually. Differential costs were applied to patients experiencing ESLD complications depending on the duration of the complication, i.e. different costs were applied in the first year of the disease state compared to subsequent years. Similarly, each health state was subject to specific health-related quality of life estimates applied annually. Default disease state costs and health utilities, specific to the UK, are presented in S1 Table.

### Life expectancy, quality-adjusted life expectancy and disease management cost

It is conventional in health economic analyses to compare the projected incremental costs and health benefits associated with the efficacy and safety profiles of each treatment alongside the relevant therapy-specific costs and disutilities. While the MONARCH model is designed to undertake such analyses, the principle objective of this study was to evaluate the expected benefits associated varying levels of SVR, independent of the therapy responsible for achieving it. The rationale for this approach was that, with the current expansion of therapeutic choice in HCV combined with the complexity associated with the interpretation of a conventional incremental cost-effectiveness analysis, the benefits associated with the primary objective of treatment (SVR), rather than the benefit of any particular regimen, can be observed. LY gains, QALY gains and complication management costs were estimated over a lifetime for patients currently aged 40, 50 and 60 years in fibrosis stages F0–F4 for various plausible SVR rates. The SVR rates chosen were 0% (representing no treatment) and 10% increases in SVR between 40% and 100%, representing a range of SVR rates achievable though treatments such as peg-interferon alfa plus ribavirin, triple therapy with telaprevir or boceprevir and the emerging DAAs [22–30]. Projections of LYs, QALYs and complication management costs were obtained using the fibrosis stage transition rates described in Table 2. For this analysis, it was assumed that 41% of patients had contracted HCV through intravenous drug use (IDU) and 31% through blood transfusions, with an average duration of infection of 17.5 years; 17.5% were assumed to consume excess alcohol. These assumptions were based on the average profile of HCV patients within the Thein *et al*. study [16]. It was also assumed that 44.6% of individuals were infected with HCV genotype 1 [16].

LYs, QALYs and costs were discounted at 3.5%. The need to apply an annual discount to a present value is widely accepted in economic evaluation. An annual discounting rate of 3.5% is applied to both costs and health effects, based on guidance from the National Institute for Health and Care Excellence (NICE) [31]. Both discounted (where an annual rate of 3.5% has been applied) and non-discounted results (where the annual discounting rate is not applied) are presented in this analysis.

### Validation

A model validation exercise was carried out to demonstrate that the projections obtained from the MONARCH model were consistent with those previously reported, from a UK perspective. A previously published systematic literature review was used as the basis of identifying global HCV cost-effectiveness studies [32]. Within the review, nine of the studies were identified as being UK-specific; of these, two were excluded due to a lack of reported data [33,34] and a further study [14] was excluded due to its analysis being carried out using the MONARCH model. In addition, the health technology assessments (HTAs) for telaprevir [35] and boceprevir [8], licensed in combination with peg-interferon-alfa and ribavirin for the treatment of genotype 1 patients, were also incorporated in the validation, providing a total of eight cost-effectiveness studies.

The MONARCH model was first populated using data from each individual cost-effectiveness study. Where necessary data were not reported, the MONARCH model’s default parameters were used. Key assumptions regarding the reproduction of each study are presented in Table 3. Validation was also undertaken utilising the disease transition rates hard-coded within the MONARCH model to assess the generalisability of such rates. The primary study endpoints validated were total predicted costs, total QALYs, the incremental cost effectiveness ratios (ICERs) and ESLD complication incidence. ICER values were reported in all eight of the cost-effectiveness studies, whilst only two of the studies presented clinical event outcomes. The coefficient of determination, R^2^, was used as a measure of goodness of fit.

**Table 3:** Modelling assumptions utilised when populating the MONARCH model with study-specific input data.

Study	Assumptions/Notes	Reference
Cost-effectiveness studies		
Martin 2012	Excluded	[45]
Grischenko 2009	Mild, moderate and severe disease states: assumed mild patients equally distributed across F0 and F1 stages, moderate patients distributed across F2 and F3 stages and severe patients are stage F4. Assumed no subsequent cost for treatment	[38]
Grieve 2002	Mild, moderate and severe disease states: assumed mild patients equally distributed across F0 and F1 stages, moderate patients distributed across F2 and F3 stages and severe patients are stage F4. No information provided on certain complication transitions/utilities. MONARCH static rates/utilities utilised.	[39]
Grieve 2006	Mild, moderate and severe disease states: assumed mild patients equally distributed across F0 and F1 stages, moderate patients distributed across F2 and F3 stages and severe patients are stage F4. No information provided on certain complication transitions/utilities. MONARCH static rates/utilities utilised. Assumed post SVR cost was applied for 1 year. No comparator arm.	[43]
McEwan 2013	Excluded	[14]
Stein 2002	Excluded	[33]
Shepherd 2000	Chronic HCV state: assumed patients equally distributed amongst F0–F3. MONARCH transition rate utilised to model liver transplant to death. Costs of DC assumed stratified by rates used in other papers. Assumed cost of 24 weeks monitoring was half of 48 week cost.	[41]
Shepherd 2004	Chronic HCV state: assumed patients equally distributed amongst F0–F3. MONARCH transition rate utilised to model liver transplant to death. Costs of DC assumed stratified by rates used in other papers. Assumed cost of 24 weeks monitoring was half of 48 week cost.	[42]
Shepherd 2007	Mild, moderate and severe disease states: assumed mild patients equally distributed across F0 and F1 stages, moderate patients distributed across F2 and F3 stages and severe patients are stage F4. Assumed cost of 24 weeks monitoring was half of 48 week cost.	[44]
Telaprevir HTA	Mild, moderate and severe disease stages: assumed mild patients equally distributed across F0 and F1 stages, moderate patients distributed across F2 and F3 stages and severe patients are stage F4. Created average age and average disease transition rates subject to age group information provided.	[9]
Boceprevir HTA	Assumptions regarding the stratification of discontinuation and adverse events.	[8]
Epidemiological studies		
Harris 2002	Genotype stratification not reported. Assumption made regarding proportion consuming excess alcohol (assumed 13.6% as read from graph). Analysis was carried out with age sampled from a gamma distribution and with patient follow-up and genotype selection sampled from a uniform distribution.	[36]
Harris 2006		[37]

In order to assess the ability of the model’s natural history progression to predict real-world outcomes, the model was further validated to two epidemiology studies [36,37], contrasting mortality incidence. Patient characteristics that influence disease progression were selected from the studies. Due to the range of reported cohort characteristics, specifically surrounding age, follow-up and genotype, a probabilistic sensitivity analysis was conducted, simulating 1,000 patients over 1,000 simulations. Certain patient characteristics were kept constant: proportion male, proportion consuming excess alcohol and proportion who reported IDU and/or blood transfusion as the likely source of infection. Age, follow-up and proportion genotype 1 were sampled within the reported ranges, utilising normal distributions for continuous variables and beta distributions for proportions. Predicted liver-related and all-cause mortality incidence rates were compared.

## Results

**Fig 2:**
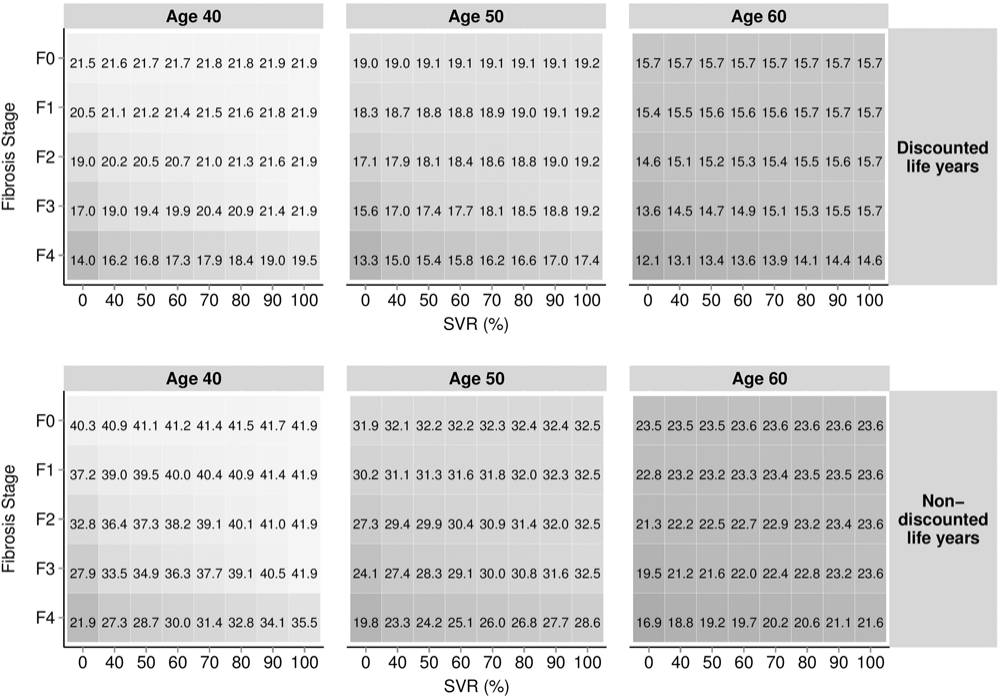
Estimated per-patient life expectancy stratified by age, discounting, current fibrosis stage and SVR.

Fig. 2 reports the per-patient discounted and non-discounted LYs for patients aged 40, 50 and 60 years, stratified by fibrosis stage (F0–F4) and rates of SVR (0%–100%). In patients with fibrosis stage F4, aged 40 years, discounted LYs ranged from 14.0 years for no treatment (0% SVR), to 19.5 with a therapy that achieved 100% SVR. Assuming no discounting, the range was 21.9–35.5 LYs. This figure also enabled the calculation of incremental LYs gained; for example, patients aged 50 in fibrosis stage F3 were estimated to gain an additional 1.8 discounted LYs when comparing treatments with an SVR rate of 100% versus 50% (19.2 versus 17.4 LYs), and 3.6 discounted LYs when comparing an SVR of 100% to no treatment (0% SVR) (19.2 versus 13.3 LYs).

**Fig 3:**
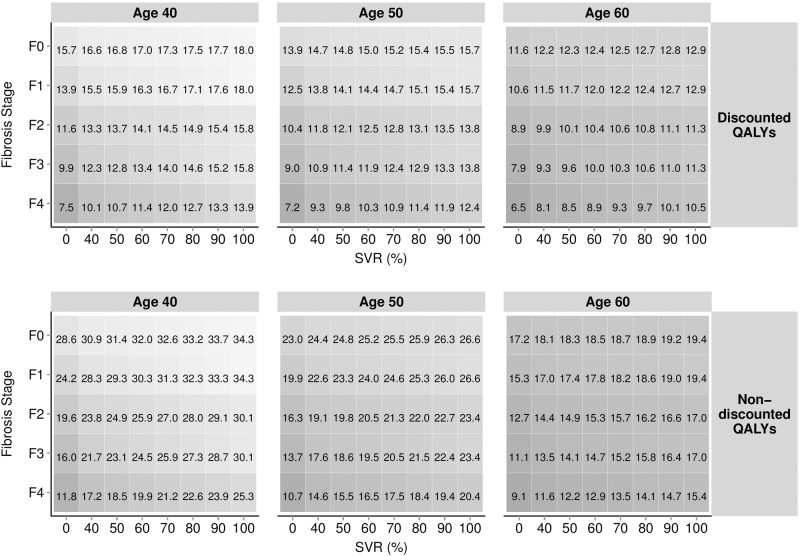
Estimated per-patient QALYs stratified by age, discounting, current fibrosis stage and SVR.

Fig. 3 depicts the graphical representation of the per-patient QALYs gained for the same scenarios (patients aged 40, 50 and 60 years, stratified by fibrosis stage [F0–F4] and rates of SVR [0%–100%]). Predicted gain in QALYs per 10% improvement in SVR varied by fibrosis stage and age, and were obtained by subtracting relevant absolute levels of QALYs reported, Fig. 3. In patients aged 40, QALY gains per 10% improvement in SVR ranged from 0.23 (F0) to 0.64 (F4) (discounted) and 0.58 (F0) to 1.35 (F4) (non-discounted). QALY gains decreased with advancing age; for example, a 10% improvement in SVR in F4 was associated with a QALY gain of 0.40 (discounted) and 0.63 (non-discounted) for 60 year old patients compared to 0.64 (discounted) and 1.35 (non-discounted) for 40 year old patients. In those aged 40, QALY were maximised when SVR reached 100%, at 18.0 years (discounted) and 34.3 years (non-discounted). QALYs decreased to 7.5 years (discounted) and 11.8 years (non-discounted) for patients in F4 who remain untreated (0% SVR). Fig. 3 also enabled the incremental QALY to be calculated for differing rates of SVR. For example, patients aged 50 in fibrosis stage F3 had an expected discounted QALY gain of 1.9 QALYs when comparing an SVR rate of 90% (3.3 QALYs) to 50% (1.9 QALYs).

**Fig 4:**
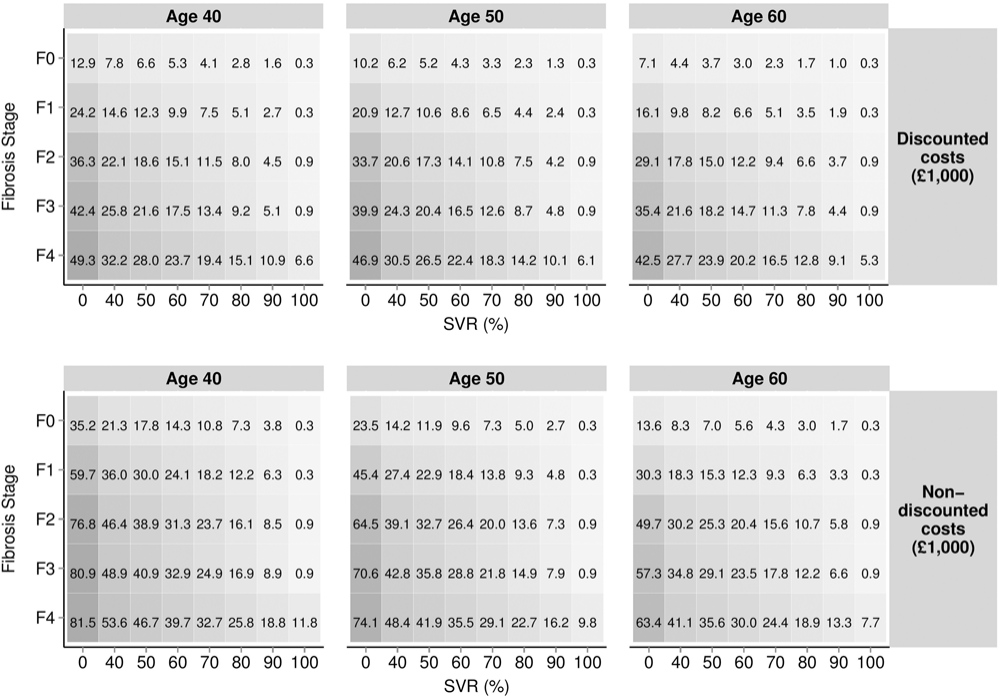
Estimated per patient costs (£000s) related specifically to HCV related complications (excluding any HCV therapy costs) stratified by age, discounting, fibrosis stage and SVR.

Fig. 4 presents the total expected per-patient lifetime discounted and undiscounted costs (£000s) associated with HCV-related complications. Projected expenditure was minimised with 100% SVR at approximately £300 when considering both discounted and non-discounted values in those aged 40, in fibrosis stage F0 and F1. Expenditure rises to £49,300 (discounted) and £81,500 (non-discounted) when considering a 40 year old patient in F4 who remains untreated (0% SVR). The incremental cost of disease-related complications for varying rates of SVR can also be calculated by subtracting absolute values; for example, within the discounted estimates, there is an expected incremental complication-related cost of £13,100 when comparing an SVR rate of 40% (total cost £20,600) with 80% (total cost £7,500) in patients aged 50 in fibrosis stage F2.

### Model validation to UK studies

**Fig 5:**
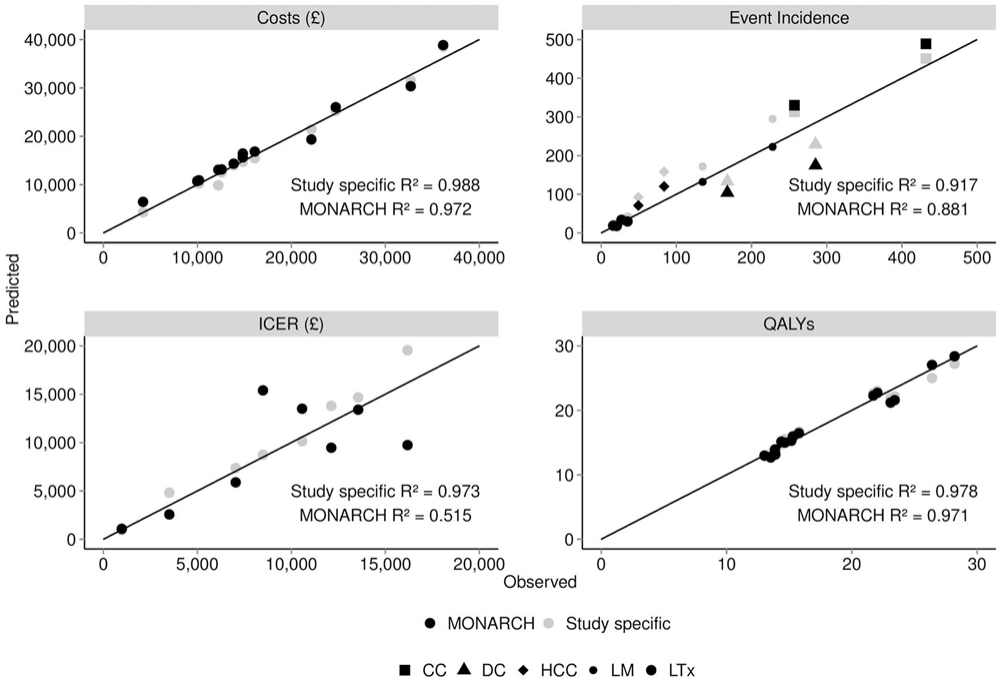
Predicted ICER values utilizing the MONARCH cost-effectiveness model compared to original study-specific ICER values as reported in 8 UK cost-effectiveness studies [8,9,38,39,41–44].

**Fig 6:**
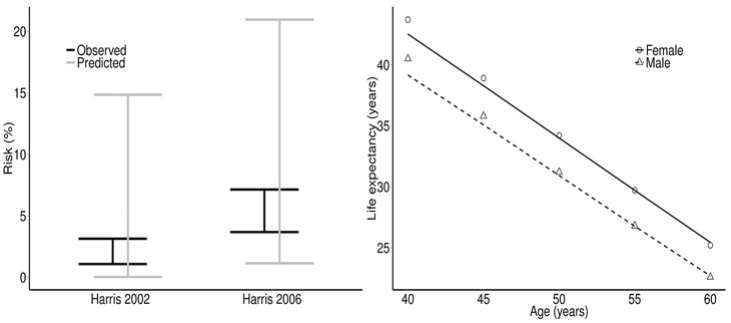
Predicted rates of liver-related mortality (validation to [26, 27]) (left) and all-cause mortality (UK life tables) (right) as estimated by the MONARCH model.

Across all cost effectiveness studies, the total costs, QALYs and ICERs from the MONARCH model, as compared to the published studies, produced R^2^ statistics of 0.988, 0.978 and 0.973, respectively, when using transition rates from the published studies. Using default MONARCH model transition rates, R^2^ statistics of 0.972, 0.971 and 0.515 were observed, respectively (Fig. 5). The low ICER R^2^ value was due to two outliers [38,39] and highlights the challenge of validating to ratios, as both total costs and QALYs were predicted with reasonable accuracy; an R^2^ of 0.879 was obtained when omitting these two studies. Validating to clinical endpoints and comparing to the predicted number of events in published studies, produced R^2^ statistics of 0.917 and 0.881, for study-specific and MONARCH transition rates, respectively (Fig. 5). Utilising MONARCH-specific transition rates, the model produced liver mortality incidence ranges of 0.02–0.041 and 0.061–0.095 compared to observed ranges of 0.011–0.031 and 0.037–0.071, when validating to 2002 and 2006 studies, respectively. Fig. 6 depicts these results graphically alongside the results of the fit to all-cause mortality.

## Discussion

Projecting the long-term clinical and cost consequences associated with HCV disease progression is a necessary requirement for the evaluation of new technologies for the treatment of chronic HCV. The principle analysis herein demonstrates the significant impact on expected costs, LYs and QALYs associated with increasing levels of SVR. The SVR rates presented in this analysis are a realistic representation of those relevant to contemporary HCV-related clinical practice. A noteworthy observation relates to the costs and life expectancy associated with no treatment (SVR 0%), in which discounted per-patient lifetime costs approach £50,000 in those with more advanced disease and discounted life expectancy is reduced by around 8 years. With an estimated prevalence of chronic HCV infection in the UK of approximately 215,000 and around 3% treated each year [2], the potential total cost impact and life years lost is significant.

In line with reported progression rates, patients treated successfully from F4 were allowed to progress to HCC and DC [17]. This significantly impacted predicted costs and resulted in lower quality-adjusted life expectancy. Consequently it is likely that health benefit is maximised and costs minimised by treating patients prior to reaching fibrosis stage F4.

The estimated costs and health outcomes presented in this study are based on long-term modelled projections and therefore demonstrating the validity of the model used is crucial. Model validation has been described as the “process of determining whether a simulation model is an accurate representation of the system for the particular objectives of the study” [40]. Consequently, this validation exercise has focused on demonstrating that the model predicts cost-effectiveness and incidence of ESLD endpoints consistent with those previously reported in UK-specific cost-effectiveness analyses. Costs and health-related quality of life are key drivers of cost-effectiveness. Most data for costs and utilities from identified studies were transparent and readily accessible for extraction. In most studies, no cost was reported for the SVR health state beyond the drug acquisition costs and sometimes monitoring costs directly associated with treatment. Although costs and utilities for most disease complications were well documented for the first year, there were differences of approach for subsequent years spent in the same health state, with the common assumption that each year with a particular complication is associated with the same costs and utilities as the previous year(s), while other studies presented different cost and/or utility for subsequent years. The greatest variation in cost was associated with the cost of transplantation in the first year, with costs ranging from £27,330 to £58,736.

Large variability in values employed for disease transition progression rates, utilities and costs can impact cost-effectiveness analyses. This was most notable when validating ICER values using MONARCH transition rates: ICER values were over- and under-estimated when validated to studies by Grieve *et al*. [39] and Shepherd *et al*. [41]. The study by Grieve *et al*. [39] is an economic evaluation of interferon-alfa in mild chronic hepatitis C patients. The likely cause of over-estimation is the effect of slower disease progression with MONARCH transition rates compared to rates reported in Grieve *et al*. [39]. Assuming no treatment intervention, the 20-year incidence rate of ESLD complications for patients in F4 (compensated cirrhosis) observed with MONARCH was 0.120 compared with an incidence of 0.309 for the Grieve study. This absolute difference caused an underestimation of the QALY gain between treatment arms. The second study, by Shepard *et al*. [41], was a HTA for the use of interferon-based dual therapy in the treatment of chronic hepatitis C. The study progressed patients from a single state of chronic hepatitis C to ELSD complications. In order to replicate the study using MONARCH transition rates, there was a need to distribute patients across fibrosis stages, since no information was available regarding fibrosis severity; a uniform distribution across fibrosis stages (F0–F3) was assumed. This assumption led to a significantly increased rate of progression to ESLD complications; the 20-year incidence of F4 (compensated cirrhosis), assuming no treatment, was 0.368 when utilising MONARCH rates and 0.171 when utilising study-specific rates. This under-estimation highlights the importance of understanding baseline characteristics amongst patient cohorts.

Validation to clinical endpoints observed in UK cost-effectiveness studies provides insight into the predictive consistency of the MONARCH model to previously published analysis. Of note, however, is the relative paucity of robust epidemiological evidence suitable for informing and validating HCV disease models in general. A review of key model parameters utilised in HCV cost-effectiveness analyses identified 34 models that drew their disease progression inputs predominantly from nine studies (published between 1989–1997) [32]. Consequently, while this study demonstrates consistency with other published UK cost-effectiveness analysis, there is a need for more contemporary UK HCV epidemiological analysis to be undertaken and published.

In reviewing published UK cost-effectiveness analysis, a common pathway of six disease states was identified: chronic hepatitis C, compensated cirrhosis, decompensated cirrhosis, hepatocellular carcinoma, liver transplant and death. In some studies, the early disease states and/or the decompensated cirrhosis state were further subdivided e.g. decompensated cirrhosis was further subdivided into ascites, variceal haemorrhage and hepatic encephalopathy [41,42]. Some studies only describe chronic hepatitis C disease states as mild, moderate or severe [35,38,39,43,44]. In this instance, assumptions were made that equated mild, moderate or severe health states to fibrosis stages (mild = F0 and F1, moderate = F2 and F3 and severe = F4). These assumptions and variations between studies affected the R^2^ goodness of fit statistics.

Validating the transition rates embedded within the MONARCH model to the limited UK-specific epidemiological studies available provides reassurance that the MONARCH cost-effectiveness model has the ability to consistently model the natural history of HCV in line with other studies. An important component of model validation within the context of decision-making is the ability to demonstrate whether the choice of model used might influence the decisions made. The analysis presented here shows that use of the MONARCH model would not have significantly altered the ICERs to such an extent that it would have resulted in a different decision. This study has however highlighted those structural assumptions that can substantially influence cost-effectiveness ratios.

As the treatment landscape continues to evolve, we anticipate that the research questions of the future will begin to inform on aspects of patient heterogeneity currently unaccounted for in HCV clinical and epidemiological studies. This may necessitate a move away from a Markov modelling approach and introduce the use of patient-level simulation. In addition, greater emphasis should be placed on justifying the choice of underlying data and ensuring that data used are the most relevant to the research question. While acknowledging these data limitations, the model produces consistent estimates of life expectancy and quality-adjusted life expectancy to published UK studies and consistent with those used to inform prior HTA decisions.

## Conclusion

In this study, expected disease costs, life expectancy and quality-adjusted life expectancy for various ages, fibrosis stages and SVR rates have been tabulated. As new treatments for HCV emerge, it is useful to gauge the expected health economic consequences associated with SVR rates, without the need to employ a particular model. These tables are intended to provide a general overview of the likely benefits associated with treatment and as a consequence of improving SVR rates. The validation analysis undertaken helps to demonstrate the structural validity of the MONARCH cost-effectiveness model in projecting the long-term clinical and cost consequences associated with HCV disease progression, against a range of cost-effectiveness and clinical endpoints. The aim of the validation analysis was to provide reassurance that the projections from the MONARCH were consistent with those previously published in the UK.

## Supporting Information

S1 Table Disease state costs and health utility estimates. (DOCX)
