# Supplementary material for: Estimating the Clinical and Economic Benefit Associated with Incremental Improvements in Sustained Virologic Response in Chronic Hepatitis C
Source: PLoS One. 2015 Jan 30;10(1):e0117334. doi: 10.1371/journal.pone.0117334 (PMC4311930; doi:10.1371/journal.pone.0117334)
Supplement: S1 Table — (DOCX) [file pone.0117334.s001.docx]

**Supplementary material**

| **Table S1. Disease state costs and health utility estimates** | | | | |
| --- | --- | --- | --- | --- |
| **Disease State** | **Annual cost (£)** | **Source** | **Health utility** | **Source** |
| F0/F1 | 177 | [[40](#_ENREF_40)] | 0.77 | [[40](#_ENREF_40)] |
| F2/F3 | 922 | [[40](#_ENREF_40)] | 0.66 | [[40](#_ENREF_40)] |
| F4 | 1,464 | [[40](#_ENREF_40)] | 0.55 | [[40](#_ENREF_40)] |
| DC | 11,729 | [[40](#_ENREF_40)] | 0.45 | [[40](#_ENREF_40)] |
| HCC | 10,452 | [[40](#_ENREF_40)] | 0.45 | [[40](#_ENREF_40)] |
| LTx (year 1) | 47,311 | [[40](#_ENREF_40)] | 0.45 | [[40](#_ENREF_40)] |
| LTx (subsequent years) | 1,781 | [[40](#_ENREF_40)] | 0.67 | [[40](#_ENREF_40)] |
| SVR from F0/F1* | 333 | [[40](#_ENREF_40)] | 0.82 | [[40](#_ENREF_40)] |
| SVR from F2/F3* | 922 | [[40](#_ENREF_40)] | 0.72 | [[40](#_ENREF_40)] |
| SVR from F4* | 1,464 | [[40](#_ENREF_40)] | 0.72 | ^Assumption based on [[40](#_ENREF_40)] |
| CC, compensated cirrhosis; DC, decompensated cirrhosis; HCC, hepatocellular carcinoma; LTx, liver transplant; SVR, sustained virologic response.  Notes: All costs inflated to 2012/13 values using the Hospital & community health services (HCHS) index [[41](#_ENREF_41)].  *Costs applied in the first year after SVR is achieved only, in line with previous studies [[36](#_ENREF_36),[40](#_ENREF_40)].  ^ Assumed the health utility associated with SVR from F4 is the same as the health utility associated with SVR from F2/F3. | | | | |
